# Non-Proteinuric Diabetic Nephropathy

**DOI:** 10.3390/jcm4091761

**Published:** 2015-09-07

**Authors:** Nicolas Roberto Robles, Juan Villa, Roman Hernandez Gallego

**Affiliations:** 1 Cátedra de Riesgo Vascular, Facultad de Medicina, Universidad de Salamanca, Salamanca 37007, Spain; 2 Servicio de Nefrologia, Hospital Infanta Cristina, Badajoz 06070, Spain; E-Mails: jvilla_19@hotmail.com (J.V.); romanhg78@hotmail.com (R.H.G.)

**Keywords:** chronic kidney disease, proteinuria, microalbuminuria, diabetes mellitus

## Abstract

Diabetic nephropathy patients traditionally show significant macroalbuminuria prior to the development of renal impairment. However, this clinical paradigm has recently been questioned. Epidemiological surveys confirm that chronic kidney disease (CKD) diagnosed by a low glomerular filtration rate (GFR) is more common in diabetic patients than in the non-diabetic population but a low number of patients had levels of proteinuria above that which traditionally defines overt diabetic nephropathy (>500 mg/g). The large number of patients with low levels of proteinuria suggests that the traditional clinical paradigm of overt diabetic nephropathy is changing since it does not seem to be the underlying renal lesion in most of diabetic subjects with CKD.

## 1. Introduction

An impaired glomerular filtration rate (GFR) is the final common pathway of kidney disease. Once the GFR is impaired, progression to end-stage renal disease (ESRD) and cardiovascular disease occur even with appropriate medical management.

Prevalence of diabetes has reached epidemic proportions in the world. According to the International Diabetes Federation, there were 366 million people with diabetes in 2011, and this figure is expected to increase above 500 million by 2030 [1]. Most people with diabetes do not live in high income countries, and these countries shall have the greatest increase in diabetes over the next decades. Along with the global epidemic of diabetes, diabetic nephropathy has become a major public health and clinical problem all over the world. Using data from the NHANES (National Health and Nutrition Examination Survey) it has been estimated the disease burden of diabetic nephropathy in the US adult population aged 20 years or older. Diabetic nephropathy was defined as diabetes with the presence of albuminuria, reduced GFR, or both. The prevalence of diabetic nephropathy was 3.3%. Among the diabetic adults, the prevalence of nephropathy was 34.5%, the prevalence of impaired GFR (with or without albuminuria) was 17.7% and the prevalence of albuminuria (with or without reduced GFR) was even higher (23.7%) [2]. In the same way, Parving *et al.* reported the prevalence of albuminuria in a cross sectional survey among more than 30,000 Type 2 diabetes patients from 33 countries [3]. The overall prevalence of microalbuminuria was 38.8% and macroalbuminuria was 9.8% with wide racial differences. However, only 22% of patients had impaired renal function (GFR < 60 mL/min).

The prevalence of diabetic nephropathy has been increasing all over the world. For example, in USA the diabetic nephropathy prevalence raised 34% from 1988–1994 to 2005–2008 [2] due to the rising prevalence of diabetes mellitus, without a change in the prevalence of diabetic nephropathy among those with diabetes. Increases in diabetic nephropathy prevalence were largest for elderly people among whom diabetic nephropathy was most common though this increase might be partially due to increased diagnostic surveillance. Currently diabetic nephropathy is the single leading cause of starting renal replacement therapy, accounting for nearly half of all ESRD cases in the US population [4] as well as in other western populations [5,6].

## 2. Classic Diabetic Nephropathy

Although much progress has been made in slowing the progression of diabetic nephropathy, chronic kidney failure and development of ESRD remain frequent in diabetes and diabetic nephropathy continues to be the most common cause of ESRD in developed nations [7]. Typically, patients proceed through five stages, *i.e.*, glomerular hypertrophy and hyperfiltration, renal structural changes without microalbuminuria, microalbuminuria with preserved renal function, significant proteinuria (overt nephropathy) and progressive renal impairment and finally, the development of ESRD [8,9]. Overt nephropathy is characterized by persistent macroalbuminuria (>300 mg/24 h or mg/g) that usually precedes a decrease in GFR. Thereafter, proteinuria tends to increase and GFR starts a non-stop decline. Significant proteinuria has therefore usually been regarded as the hallmark of diabetic nephropathy (DN) when diabetic retinopathy has been diagnosed [10]. Nowadays, renal biopsy is indicated only on diabetic patients under the suspicion of the presence of nephropathies other than DN. However, there are no standardized criteria for performing kidney biopsy in diabetic patients. Absence of retinopathy, presence of hematuria, rapid onset of macroalbumiuria, active urinary sediment, rapid decrease of GFR, and suspicion of other nephropathies secondary to systemic disease, are some of the indications for renal biopsy [11].

The natural history of diabetic nephropathy has mainly been studied in Type 1 diabetes, since in Type 1 diabetic patients, the time of onset of diabetic disease is usually known. For patients with Type 1 diabetes with a duration longer than five years, the presence of sustained microalbuminuria is typically associated with the development of diabetic nephropathy [12]. In 20%–30% of Type 1 diabetic patients persistent microalbuminuria appears within the first 15 years of diabetes [13]. Microalbuminuria precedes macroalbuminuria (>300 mg of urinary albumin excretion per day) in both Type 1 and Type 2 diabetes. Renal endpoints (ESRD or doubling of serum creatinine) generally occur within ten years in approximately 20% of microalbuminuric patients, but in 60% of macroalbuminuric patients [14,15].

Histologically, diabetic nephropathy is characterized by glomerular basement membrane thickening, diffuse mesangial sclerosis with nodular formation (Kimmelstiel-Wilson lesion) as well as hyalinosis, microaneurysms and a hyaline arteriosclerosis of renal vessels [16]. Biopsies diagnosed as diabetic nephropathy are classified as follows:

- **Class I**, glomerular basement membrane thickening: isolated glomerular basement membrane thickening and only mild, nonspecific changes by light microscopy that do not meet the criteria of classes II through IV.
- **Class II**, mesangial expansion, mild (IIa) or severe (IIb): glomeruli classified as mild or severe mesangial expansion but without nodular sclerosis (Kimmelstiel-Wilson lesions) or global glomerulosclerosis in more than 50% of glomeruli.
- **Class III**, nodular sclerosis (Kimmelstiel-Wilson lesions): at least one glomerulus with nodular increase in mesangial matrix (Kimmelstiel-Wilson) without changes described in class IV.
- **Class IV**, advanced diabetic glomerulosclerosis: more than 50% global glomerulosclerosis with other clinical or pathologic evidence that sclerosis is attributable to diabetic nephropathy [17].

An accurate estimate of damage in DN can only be achieved by the histological analysis of biopsy samples. Renal vasodilation and hyperfiltration occur early in the onset of diabetes mellitus prior to any kidney damage. During the second stage, morphologic lesions develop before signs of clinical disease are present. The earliest structural abnormality in diabetes is glomerular basement membrane thickening. The kidney with early diabetes suffers significant hypertrophy; characterized by enlargement of the organ with a combination of hyperplasia and hypertrophy which is surprisingly often observed at the time of diabetes diagnosis [18]. Mesangial expansion and occlusion of glomerular capillaries lead to a loss of available surface area for filtration and to a decline in function; this third stage (also named incipient nephropathy) is characterized by microalbuminuria [9]. Persistent increased urinary albumin excretion develops most frequently during the second decade after diagnosis of diabetes and it reflects the existence of endothelial damage in the absence of specific renal lesions; and it is also associated with the beginning of advanced podocyte loss [19]. Overt nephropathy is characterized by persistent proteinuria which usually accompanies a reduction in GFR and is associated with the typical diabetic kidney lesions.

## 3. Non-Proteinuric Diabetic Kidney Disease

Despite this clearly defined pathway, there have been increasing reports that in both Type 1 and Type 2 diabetes, a proportion of patients may have renal impairment without significant proteinuria or albuminuria, with a variable percentage of patients in these reports having advanced (stages 3–5) kidney disease. Among 301 Type 2 diabetic patients attending an outpatient clinic in Australia [20] and 1197 patients from the Third National Health and Nutrition Examination Survey (NHANES III) [21,22], of patients with GFR less than 60 mL/min, 39% and 36%, respectively, were normoalbuminuric. Recently, a nonalbuminuric renal impairment syndrome was described in Type 2 diabetic patients, which has distinct clinical features that are not clearly associated with poor glycemic control and that are correlated less closely with retinopathy and high blood pressure [23]. This entity is associated with a higher prevalence of cardiovascular diseases (CVD); therefore a predominance of macroangiopathy as the underlying renal pathology has been suggested, which has yet to be demonstrated. Similar results were reported by the National Evaluation of the Frequency of Renal impairment cOexisting with NIDDM (NEFRON) 11, an incident-driven survey of 3893 patients with Type 2 diabetes in the primary care setting, has suggested that nonalbuminuric renal impairment has become the predominant form of stage 3 CKD [24], the UK Prospective Diabetes Study (UKPDS) [25] and the Action in Diabetes and Vascular disease: preterAx and diamicroN-MR Controlled Evaluation trial [26]. Obviously, since there is not proteinuria in these patients, GFR estimation is likely related to the increased diagnosis of chronic kidney disease in diabetic subjects. In fact, it has been suggested that normoalbuminuric diabetic patients might represent an artificial group, stemming from overdiagnosed CKD due to the underestimation of high GFR by the MDRD equation. Nevertheless, studies using isotopic determination of GFR argue against this hypothesis: although the MDRD underestimated the GFR, the difference was slight and not significant. The proportion of subjects for which isotopic GFR was <60 mL/min per 1.73 m^2^ was about the half in the normoalbuminuric group than in the normoalbuminuric, and the correlation between estimated and measured GFR was better in the normoalbuminuric group [27].

### 3.1. Histopathological Findings

The Tervaert classification [28] has been questioned in the basis of an exceeding importance of glomerular lesions. Some studies suggest a high incidence of normal glomerular structure among microalbuminuric and proteinuric Japanese Type 2 diabetic patients [17]. A study of a large group of Caucasian Type 2 diabetic patients showed that several had normal glomerular structure despite persistent microalbuminuria or macroalbuminuria, even though diabetic glomerular structural parameters were more altered on average from normoalbuminuria to microalbuminuria and overt proteinuria. Moreover, diabetic glomerulopathy was less apparent in patients with Type 2 diabetes than in those with Type 1 diabetes and similar renal function [29]. In Type 1 diabetes, the most important structural changes involve the glomerulus predominantly, whereas light microscopy studies have shown that a substantial proportion of Type 2 diabetic patients have more advanced tubulo-interstitial and vascular than glomerular lesions [30,31].

On this basis, a different classification system for renal lesions in diabetic nephropathy has been proposed. It includes three major groups [32]:

- **Normal or near-normal renal structure**. These patients (41%) had biopsies which were normal or showed mild mesangial expansion, tubulo-interstitial changes, or arteriolar hyalinosis.
- **Typical diabetic nephropathology**. These patients (26%) had established diabetic lesions with an approximately balanced severity of glomerular, tubulo-interstitial, and arteriolar changes. This picture was typical of that seen in Type 1 diabetic patients with obvious light microscopy DN changes.
- **Atypical patterns of renal injury.** These patients (33%) had relatively mild glomerular diabetic changes despite disproportionately severe renal structural changes in all possible combinations:
  **a.**  Tubular atrophy, tubular basement membrane thickening, and reduplication and interstitial fibrosis (tubulo-interstitial lesions).
  **b.**  Advanced glomerular arteriolar hyalinosis commonly associated with atherosclerosis of larger vessels.
  **c.**  Global glomerular sclerosis.

Dalla Vestra *et al.* have investigated renal structure in the early stages of nephropathy (microalbuminuria) in patients with Type 1 and Type 2 diabetes. Diabetic glomerulopathy was quite advanced in Type 1 diabetic patients with microabuminuria. Early diabetic glomerulopathy was detected by electron microscopy in normoalbuminuric (NA) patients and found to be more advanced in those with microalbuminuric (MA) and proteinuria. However, lesions were milder than in Type 1 diabetic patients, and there was considerable overlap between groups. Morphometric results by electron microscopy were similar to those by light microscopy, demonstrating the heterogeneity of renal structure in Type 2 diabetic patients. In fact, only 30% of MA patients had typical diabetic glomerulopathy, while 40% had more advanced tubulo-interstitial and/or vascular lesions and 30% had normal renal structure [33].

Caramori *et al*. studied a group of 105 normoalbuminuric Type 1 diabetic patients with at least 10 years of diabetes duration that had a renal biopsy performed. Patients were divided according to GFR into groups with normal (≥90 mL/min) or reduced (<90 mL/min) GFR [34]. Clinical and renal structural parameters were compared between these two groups. Glomerular structural parameters were estimated by electron microscopic morphometry. A bit more than 20% of normoalbuminuric diabetic patients had an estimated GFR < 90 mL/min). These 23 patients with reduced GFR had more advanced glomerular lesions (see Table 1) but no Kimmelstiel-Wilson nodules were described nor were sclerotic lesions. Compared with control values, glomerular basal membrane width was increased by 42% in the normal and by 64% in the low GFR groups. Similarly, the mesangial part of glomerulus was increased by 40% in the normal and by 70% in the low GFR groups, whereas mesangial matrix was increased by 67% in the normal and by 122% in the low GFR groups. Surface density of the peripheral glomerular basal membrane (GBM) per glomerulus was significantly decreased in both normal (by 8%) and low (by 25%) GFR groups. The finding of reduced GFR was much more common among female patients, particularly if retinopathy and/or hypertension were also present. This report confirms that reduced GFR occurs among long-standing normoalbuminuric Type 1 diabetic patients and is associated with more advanced diabetic glomerular lesions and, probably, with increased risk of progression [34]. Once again, glomerular lesions seems to be more common in Type 1 diabetic patients.

**Table 1:** Glomerular structural characteristics in normoalbuminuric Type 1 diabetic patients compared with nondiabetic control subjects (modified from Caramori *et al.* [34]).

	CONTROL	GFR > 90	GFR < 90
GBM width	331.5 ± 45.7	469.4 ± 84.2	544.5 ± 140.7
Sv (PGBM/glom)	0.20 ± 0.03	0.28 ± 0.06	0.34 ± 0.08
Vv (MC/glom)	0.09 ± 0.02	0.15 ± 0.04	0.20 ± 0.06
Vv (Mes/glom)	0.08 ± 0.02	0.08 ± 0.02	0.10 ± 0.02
Vv (MM/glom)	0.126 ± 0.018	0.116 ± 0.019	0.094 ± 0.021

GFR, glomerular filtration rate; GBM, glomerular basal membrane; PGBM, peripheral GBM; MC, mesangial cell; Mes, mesangium; MM, mesangial matrix; Sv (PGBM/glom), surface density of the peripheral GBM per glomerulus; Vv (MC/glom), fractional volume of the glomerulus occupied by MC; Vv (Mes/glom), fractional volume of the glomerulus occupied by mesangium; Vv (MM/glom), fractional volume of the glomerulus occupied by MM.

### 3.2. Risk of Progression

Tsalamandris *et al*. described patterns of progression of albuminuria and renal function in a subgroup of 40 patients from a total cohort of 211 diabetic patients (118 Type 1, 93 Type 2) followed over a period of 8–14 years. Forty patients (18 with Type 1 diabetes, 22 with Type 2 diabetes) showed progressive increases in albuminuria and/or decreases in creatinine clearance during the study period [35]. Of these, albuminuria alone increased in 15 patients, albuminuria increased and creatinine clearance decreased in 13 patients, and renal function decreased in 12 patients without microalbuminuria, with a similar distribution of Type 1 and Type 2 diabetic patients in each group. Of the 25 patients who showed a decrease in renal function, creatinine clearance fell at an annual rate of 4–5 mL/min. The rate of fall was not related to the presence or absence of concomitant increases in albuminuria. However, a significant preponderance of women in the group showed a decline in creatinine clearance alone [35]. So that risk of renal failure progression was not different for normoalbuminuric patients and female gender was a risk factor for decreasing renal function.

Rigalleau *et al.* followed up for 38 ± 11 months a group of 89 patients with diabetes and an estimated GFR < 60 mL/min using ^51^Cr-EDTA isotopic GFR determination [27]. Of the subjects, 15 (17%) were normoalbuminuric. They were less affected by diabetic retinopathy, and their HDL cholesterol and hemoglobin were higher. None of the CKD normoalbuminuric subjects started dialysis (microalbuminuric, 5%; macroalbuminuric, 26%) or died (microalbuminuric, 8%; macroalbuminuric, 18%) during the follow-up period. In the same way, their albuminuria and serum creatinine values were stable after 38 months, whereas the urinary albumin excretion increased in the microalbuminuric patients, and the serum creatinine increased in the macroalbuminuric patients. As expected, because of normoalbuminuria and other favorable characteristics, their risk for CKD progression or death was lower.

Perkins *et al.* follow up a cohort of 109 patients who developed new-onset microalbuminuria in the first four years following enrollment. Of these, 79 patients were followed for an average of 12 years after microalbuminuria onset. The concordance between these outcomes was weak. Only 12 of the 23 patients who progressed to advanced (stages 3–5) chronic kidney disease developed proteinuria, which, in general, did not precede but accompanied the progression to advanced chronic kidney disease. The remaining 11 patients who developed advanced disease had persistent microalbuminuria or returned to normal albuminuria. Thus, they found that one-third of patients with Type 1 diabetes developed advanced chronic kidney disease relatively soon after the onset of microalbuminuria and this was not conditional on the presence of proteinuria [36].

### 3.3. Causes of Non-Proteinuric Diabetic Nephropahty

In interpreting the increasing prevalence of nonalbuminuric renal impairment it must be taken into account the impact of changes in treatment. For instance, during the past two decades, the number of diabetic patients with hypertension and/or nephropathy treated with renin-angiotensin axis blocking drugs has been dramatically increasing. In the RIACE (Renal Insufficiency and Cardiovascular Events) cohort [21], 58.1% of the patients were on such a treatment and an even higher percentage has been reported in the recent NEFRON 11 [24]. These figures are quite different from the 13% reported in the NHANES III in years 1988–1994 [22].

In addition, the increasing prevalence of nonalbuminuric chronic kidney diseases may be due to changes in the underlying pathology of renal disease in Type 2 diabetes, with macroangiopathic lesions prevailing over microangiopathic ones. Such a shift may also reflect changes in treatment, particularly to improved glycemic and control and reduced lipids levels, and blood pressure (BP) levels that is being achieved in diabetic patients. In this regard, this view is consistent with the UKPDS results showing that glycated hemoglobin (HbA1c) is an independent risk factor for increased albumin excretion, but not for GFR impairment [33]. Moreover, non-proteinuric kidney disease was less closely associated with the other microvascular complication of diabetes, *i.e*., retinopathy, than the classic proteinuric diabetic nephropathy. However, in the Atherosclerosis Risk in Communities Study, the association between HbA1c and incident CKD was present even in the absence of either albuminuria or retinopathy [37].

Hypertension correlated less strongly with non-proteinuric than with albuminuric diabetic nephropathy but, surprisingly, non-proteinuric renal impairment is significantly associated with acute major CVD events (RIACE). This is consistent with the view that GFR less than 60 mL/min is a powerful predictor of CVD morbidity and mortality in the general population and in diabetic patients, independent of traditional CVD risk factors and albuminuria [38]. Nonetheless, this could be interpreted in a rather different way. Numerous studies have documented an inverse relationship between DBP and coronary heart disease (*i.e*., a J-shaped curve). In most studies, the J-shaped curve was found to be in the physiologic range at levels of DBP below 70 to 80 mm Hg [39]. Recently, the results of a retrospective cohort study within the Kaiser Permanente Southern California health system have been reported. Among 398,419 treated hypertensive subjects, ESRD occurred in 4957 (1.2%). The nadir systolic and diastolic BP for the lowest risk was 137 and 71 mm Hg, respectively. Stratified analyses revealed that the diabetes mellitus population had a similar hazard ratio curve but a lower nadir at 131 and 69 mm Hg but age >70 had a higher nadir (140 and 70 mm Hg). Both higher and lower treated BP compared with 130 to 139 mm Hg systolic and 60 to 79 mm Hg diastolic ranges had worsened outcomes, in other words: a J curve effect for ESRD was found. In this regard, the lack of association of high blood pressure with non-proteinuric diabetic nephropathy could be related to extremely tight blood pressure control.

Superimposed renovascular disease, senile nephrosclerosis, cholesterol emboli, and concomitant additional renal disease may all help to explain this. The potential role of episodes of acute kidney injury in these patients at high risk of cardiovascular disease also needs to be elucidated. These patients are at increased risk of cardiovascular disease and as a consequence may experience repeated episodes of acute kidney injury in association with cardiovascular events or investigations. Two studies have drawn attention to the higher incidence of subsequent ESRD in patients apparently recovering from episodes of acute kidney injury [40,41]. The long-term consequences of such events on the natural history of diabetic nephropathy are yet to be established.

Drug therapy, in particular the widespread usage of agents blocking the renin-angiotensin-aldosterone system (RAAS), such as angiotensin-converting enzyme inhibitors (ACEIs) and angiotensin 2 receptor blockers (ARBs) may also be exerting an effect. Epidemiological data from all over the world point to the increasing incidence of renal failure encompassing acute kidney injury (AKI), CKD and ESRD. In the USA, between 1996 and 2003, the incidence of non-dialysis requiring community-based AKI increased from 322.7/100,000 to 522.4/100,000 person years. Equally, the incidence of dialysis-requiring AKI increased from 19.5/100,000 to 29.5/100,000 person years [42]. Hospital incidence for AKI per 10,000 population in the USA increased from 1.8 in 1980 to a shocking 36.5 in 2005. Simultaneously, in 2008, a US Centers for Disease Control and Prevention report showed an acceleration of CKD prevalence up to 16.5% among the US population in the period 1999–2004, representing a 15.9% increase in CKD prevalence when compared to the 1988–1994 period [43]. As a result of some of these foregoing themes, Onuigbo has hypothesized that RAS blocking drugs, may be responsible for some of the observed increasing CKD/ESRD/AKI incidence currently observed in the US [44,45]. There is a general assumption among physicians and some nephrologists is that the impact of AKI on renal function is usually short-lived and transient, with the typical course of an expected recovery of renal function in most instances [46]. Nevertheless, there is now growing evidence demonstrating that, quite often, much less renal recovery occurs after AKI on CKD events, and this is generally unrecognized by physicians [47]. Moreover, there is new and cumulative evidence that AKI may leads to acute yet irreversible ESRD, the so-called newly described syndrome of rapid onset end stage renal disease [48] and this has been demonstrated for diabetic patients [49]. In this regard, a subgroup analysis of the Antihypertensive and Lipid-Lowering Treatment to Prevent Heart Attack Trial (ALLHAT) diabetic population revealed that more patients in the lisinopril group progressed to ESRD compared to the chlorthalidone group—25/1563 *vs.* 26/2755 (*p* = 0.05; relative risk (RR) 1.74; 95% confidence interval (CI): 1.00–3.01) , in spite of the theoretic beneficial effects of ACEIs in diabetic kidney disease which have been discussed above. Nevertheless, in the study by Penno *et al*. [23], the prevalence of the nonalbuminuric phenotype was even higher in stage 3 CKD patients not treated with renin-angiotensin axis blocking drugs than in all individuals with reduced eGFR (63.6% *vs.* 56.6%). In fact, the use of these agents was more common in patients with than in those without albuminuria, yet this may be an indication effect. Moreover, it has been suggested that in a subgroup of predominantly elderly Type 2 diabetic patients, oversuppression of the RAS may itself be responsible for severe reductions in GFR and albuminuria [50,51]. In this way, Suissa *et al.* presented a population-based study suggesting that ACEIs do not appear to decrease the long-term risk of ESRD in diabetes. This study was based on a registry of medication prescription, including diabetic patients who were prescribed antihypertensive agents from 1982 to 1986. The 6102 patients were followed to the end of 1997 with respect to development of ESRD, which occurred in 102 patients, of whom 21 had been treated with an ACEI within the initial 3 years of follow-up. The adjusted rate ratio for renal failure was 2.5 in patients initially treated with ACEIs compared with patients treated with diuretics, when compared with the control patients [52]. Furthermore, it has been reported that stopping inhibitors of the renin-angiotensin system in patients with advanced chronic kidney disease delayed the onset of renal replacement therapy in the majority of the patients [53].

There is a paucity of histological data from diabetic patients with non-proteinuric renal impairment since these patients are not usually submitted to renal biopsy, but it is reasonable to hypothesize that age-related glomerulosclerosis, interstitial fibrosis, and ischemic vascular disease, may also be significant [26]. Cholesterol emboli might also have contributed to loss of renal function [54]. This issue is bedeviled by the fact that biopsies are not routinely performed in diabetic patients.

Last, but not least, the effect of increasing age and clustered cardiovascular risk factor on incidence and extent of non-diabetic change may be significant and it seems likely that typical age-related changes would have a higher incidence in cohorts with higher mean age and cardiovascular risk. In the Candesartan Antihypertensive Survival Evaluation in Japan Trial (Case-J) cardiovascular events showed the strongest association with renal dysfunction defined as proteinuria or a serum creatinine higher than 1.3 mg/dL. This relationship was closer than that with left ventricular hypertrophy, diabetes mellitus, age and even BP [55].

The natural history of type diabetic patients with renal impairment and low levels of proteinuria remains to be established. Whilst some authors described a benign prognosis in their non-albuminuric patients [23], others found no difference in the rate of loss of renal function between non-proteinuric and proteinuric patients [21]. Recent data published on behalf of the ADVANCE(Action in Diabetes and Vascular Disease: Preterax and Diamicron MR Controlled Evaluation) Collaborative Group have identified both high albuminuria and low GFR as independent risk factors for both cardiovascular and renal events in type 2 diabetic patients [26]. Nevertheless, the classical view is that macroalbuminuria carries greater risk of accelerated GFR loss than that associated with a reduced GFR alone [9].

Most of diabetic patients with chronic kidney disease (GFR < 60 mL/min) have no albuminuria. Therefore, classic diabetic nephropathy does not appear to be the underlying renal lesion in a substantial number of diabetic subjects with chronic kidney disease. The causes for this changing pattern of renal disease in diabetic patients remains to be settled.
